# Dynamic prediction based on variability of a longitudinal biomarker

**DOI:** 10.1186/s12874-021-01294-x

**Published:** 2021-05-15

**Authors:** Kristen R. Campbell, Rui Martins, Scott Davis, Elizabeth Juarez-Colunga

**Affiliations:** 1 Department of Pediatrics, University of Colorado Anschutz Medical Campus, Aurora, 80045 Colorado USA; 2 Centro de Estatística e Aplicações da Universidade de Lisboa (CEAUL); Faculdade de Ciências da Universidade de Lisboa, Departamento de Estatística e Investigação Operacional, Lisboa, 1749-016 Portugal; 3 Division of Renal Diseases and Hypertension, University of Colorado Anschutz Medical Campus, Aurora, 80045 Colorado USA; 4 Department of Biostatistics and Informatics, University of Colorado Anschutz Medical Campus, Aurora, 80045 Colorado USA

**Keywords:** Dynamic prediction, Kidney transplant, Survival

## Abstract

**Background:**

Tacrolimus is given post-kidney transplant to suppress the immune system, and the amount of drug in the body is measured frequently. Higher variability over time may be indicative of poor drug adherence, leading to more adverse events. It is important to account for the variation in Tacrolimus, not just the average change over time.

**Methods:**

Using data from the University of Colorado, we compare methods of assessing how the variability in Tacrolimus influences the hazard of de novo Donor Specific Antibodies (dnDSA), an early warning sign of graft failure. We compare multiple joint models in terms of fit and predictive ability. We explain that the models that account for the individual-specific variability over time have the best predictive performance. These models allowed each patient to have an individual-specific random error term in the longitudinal Tacrolimus model, and linked this to the hazard of dnDSA model.

**Results:**

The hazard for the variance and coefficient of variation (CV) loading parameter were greater than 1, indicating that higher variability of Tacrolimus had a higher hazard of dnDSA. Introducing the individual-specific variability improved the fit, leading to more accurate predictions about the individual-specific time-to-dnDSA.

**Conclusions:**

We showed that the individual’s variability in Tacrolimus is an important metric in predicting long-term adverse events in kidney transplantation. This is an important step in personalizing the dosage of TAC post-transplant to improve outcomes post-transplant.

**Supplementary Information:**

The online version contains supplementary material available at (10.1186/s12874-021-01294-x).

## Background

In many medical settings, the variability in patient drug levels is hypothesized to predict adverse events, as higher variability is often indicative of drug-drug interactions, altered drug metabolism, or poor drug adherence. This is the case in the kidney transplant setting: Tacrolimus (TAC) is prescribed post-transplant to suppress the immune system, and the amount of drug in the body is measured frequently via blood draw. In scenarios such as this, it is important to account for the variation in the longitudinal outcome, not just the average change over time. In this paper, we test whether the variability in longitudinal TAC predicts time-to-de novo Donor Specific Antibodies (dnDSA), an early warning sign of kidney transplant graft failure.

Clinicians need accurate predictions of adverse allograft events in order to know when and how to modify the treatment course for high risk patients, such as altering immunosuppression therapy or increasing follow-up visit frequencies. This is critically important today, as there exists a national shortage of available kidney donors [1]. If adverse events can be avoided, the available donated organs can be used more effectively, and in turn the kidney transplant waiting list, currently at nearly 100,000 patients [1], will be shorter. Avoiding antibody-mediated rejection, or organ rejection due to de novo donor-specific antibodies (dnDSA), is one of the most important steps in achieving long-term graft survival [2]. It has been shown that maintaining proper immunosuppression therapy levels post-transplant is a major determinant in preventing dnDSA. Tacrolimus (TAC) is the primary immunosuppressant used by U.S transplant centers (93%) [3, 4], but it’s therapeutic dose window is narrow and differs by patient.

TAC trough levels, or the lowest amount of drug in the blood prior to the next dose, are measured frequently post-transplant. In previous studies, one-dimensional functions of observed TAC values were found to be associated with dnDSA; these functions included the percentage of TAC levels below 5 ng/ml [5], average TAC troughs of less than 8 ng/ml, and the percentage of time TAC was in therapeutic range [6]. Importantly, the variability of TAC was also found to be associated with dnDSA, as high variability likely indicates inadequate drug exposure and possible non-adherence. In previous studies, the coefficient of variation (CV) of TAC, summarized over a specific range of time pre-dnDSA, was predictive of dnDSA. [7–9]. The CV is calculated as the ratio of the standard deviation to the mean, and is a way to standardize the variability of a measurement so it is not dependent on the average value [10].

To complicate the problem further, dnDSA is only screened for periodically, and much less frequently than TAC trough levels are drawn. This makes dnDSA an interval censored outcome, as the exact timing of development is unknown. The majority of studies analyzing the relationship between TAC and dnDSA treat dnDSA as right censored due to the analytic complexities associated with interval-censored outcomes. A previous study used a joint model for interval censored data that incorporated all longitudinal values of TAC and found that both average TAC and the slope of TAC over time were associated with time-to-dnDSA [4]. This approach used a linear mixed model for TAC linked by a random intercept and random slope to an interval censored parametric survival model for dnDSA. Joint models such as this have become increasingly popular as a way to simultaneously model longitudinal and time-to-event data. These approaches typically use a standard linear mixed model in combination with a survival model to assess the effect of the longitudinal outcome on the time-to-event outcome. These two model processes can be linked in a number of ways, including shared random effects, the true value, or the slope of the longitudinal trajectory. More recently, research has been done into allowing for a more flexible linking function [11, 12].

In this paper, we build upon the previously mentioned joint model for TAC and dnDSA [4] to test the hypothesis that an individual’s variability of TAC over time is associated with time-to-dnDSA. We allow for each individual’s TAC trajectory to be normally distributed with heterogeneous variances, which avoids the unlikely assumption that all individuals share a common variance. We test whether this individual-specific variability is associated with time-to-dnDSA and whether it improves the predictive ability of the model. We also assess the model’s ability to dynamically predict a patient’s probability of developing dnDSA after surviving dnDSA-free for a specified period. This is motivated by the clinical scenario where patients are seen in clinic at 6 months or 1 year-post transplant dnDSA-free, and the clinician wants to assess the probability that the patient remains dnDSA-free until a future appointment. We demonstrate how these probabilities can be calculated using the joint model, and how accurate these predictions are given the dataset at hand.

In the first section, we introduce the kidney transplant study data. Next, the joint models are formulated and applied to the motivating kidney transplantation dataset. Model estimation and prediction assessments follow, and we end with a discussion of study limitations and next steps.

### Kidney transplant study

As a motivating example, we introduce a retrospective study of patients who received a kidney transplant at the University of Colorado between 2007 and 2013. Exclusion criteria included being under 18 at the time of transplant, having a simultaneous liver and kidney transplant or islet cell transplant, having DSA pre-transplant or dnDSA within the first week post-transplant, not having any dnDSA screening post-transplant, and not receiving TAC post-transplant. This study was conducted in accordance with the Declaration of Helsinki and was approved by the ethics committee at the Colorado Multiple Institutional Review Board (COMIRB 13-3137).

Post-transplant, TAC trough levels and dnDSA screening results for each of the 538 included patients were collected for up to 7 years. Because of the unreliability of TAC and dnDSA data within the first week post-transplant, follow-up data was collected starting at day 8 post-transplant. Individuals had a median of 22 (IQR: 15-32) qualifying TAC levels, taking on values from 0 to 30 and roughly following a normal distribution. Screening for dnDSA was conducted at 1, 6, 12 months, annually, and when clinically indicated. Approximately one-third (n=181) had at least one dnDSA during follow-up, and for the purpose of this study, we focused on time-to-first dnDSA. Additional variables collected included age at transplant, race/ethnicity, and the number of Human Leucocyte Antigen (HLA) mismatches between donor and recipient. A more detailed description of the dataset and measures collected can be found elsewhere [4, 6].

We randomly split the dataset into a training (2/3, N=358) and a testing (1/3, N=180) cohort. The training cohort was used to fit all models outlined in the results, while the testing cohort was used to assess predictive performance.

## Methods

### General model formulation

Suppose there are *N* individuals each measured at *n*_*i*_ time points, *i*=1,...,*N*. Let *y*_*ij*_ be the measurement for individual *i* at time *t*_*ij*_,*j*=1,...,*n*_*i*_. We assume that given the vector of individual-specific random intercept and slope (*a*_0*i*_,*a*_1*i*_)^′^, individual outcome measures *y*_*ij*_ are independent and normally distributed with mean *μ*_*ij*_ and residual variance $\sigma ^{2}_{l}$, where

1 $$ \mu_{ij}=f(t_{ij})+\beta_{1}'x_{1i}+a_{0i}+a_{1i}t_{ij}.   $$

Some examples of *f*, the function that models the time component, include a linear trajectory, i.e. *f*(*t*_*ij*_)=*b*_0_+*b*_1_*t*_*ij*_; or b-spline basis functions, i.e. $f(t_{ij})=\sum _{k=1}^{K} \beta '_{0k} \psi _{k}(t_{ij})$, where *ψ*_*k*_(*t*_*ij*_) is the *k*^*th*^ of *K* basis functions with coefficient *β*_0*k*_ and the number of basis functions *K* depends on the degree of the splines (e.g. *p*=3) and number of inner knots (*h*=3; *K*=*p*+*h*+1). Baseline covariates are represented by *x*_1*i*_, with regression coefficients *β*1′. The random effects *a*_0*i*_,*a*_1*i*_ are distributed normally with mean zero and variance/covariance matrix

2 $$ \left[\begin{array}{l} a_{0i} \\ a_{1i} \\ \end{array}\right] \sim N\left(\begin{array}{ll} \left[\begin{array}{l} 0\\ 0 \\ \end{array}\right] \end{array}, \left[\begin{array}{cc} \sigma_{0}^{2} & \rho\sigma_{0}\sigma_{1} \\ \rho\sigma_{0}\sigma_{1} & \sigma_{1}^{2} \\ \end{array}\right]\right).  $$

Importantly, the model allows a flexible residual variance component, $\sigma ^{2}_{l}$. In this paper, we either set *l* to 1, which assumes all individuals have a common residual variance, or we set *l* to *i*, which allows all individuals to have different residual variances. This flexible variance component avoids the typical assumption that all subjects share one common residual error term, in favor of allowing the model to estimate higher or lower variance depending on the amount of variability in each subject specific trajectory.

The same *N* individuals are measured for a time to event outcome at varying time points. The outcome is interval censored, as it is only known to happen between two discrete time points and the exact timing is unknown. Let *t*_*Ri*_ be the time at which the outcome was detected and *t*_*Li*_ the time of the visit immediately preceding *t*_*Ri*_. The actual unknown time to event, *t*_*i*_, occurs in between *t*_*Li*_ and *t*_*Ri*_. The hazard for individual *i* at any time *t* can be represented by

3 $$ h_{i}(t)=h_{0}(t)\exp\left(\beta_{0}+\beta_{2}'x_{2i}+\lambda_{0} a_{0i}+\lambda_{1} a_{1i}+\lambda_{2}g_{i})\right)   $$

where *h*_0_(*t*) is the baseline hazard, which can be various flexible distributions, including parametric (typically exponential, Weibull, or gamma), piecewise constant or spline. *β*_0_ represents the fixed intercept, *x*_2*i*_ is the vector of fixed covariates, which can be different from the fixed covariates in Eq. 1, and *β*_2_ is its coefficient. Of note, we restrict the longitudinal measurements (*y*_*ij*_) such that only those occurring prior to *t*_*Li*_ are used in the joint model.

The two sub-models (1) and (3) are connected by the shared random effects (*a*_0*i*_,*a*_1*i*_)^′^, and some function of the history of the longitudinal biomarker, *g*_*i*_. This function *g*_*i*_ can be chosen based on a priori hypotheses, and should include an estimated parameter from the longitudinal model (1). In our case, *g*_*i*_ will be a function of the heterogeneous random error of the biomarker, *σ*_*i*_. The association parameters *λ*_0_,*λ*_1_, and *λ*_2_ represent the estimated risk of event dependent on the average biomarker, slope of the biomarker, and the function, *g*_*i*_.

Conditional on random effects, *a*_0*i*_,*a*_1*i*_, the log likelihood can be written for each individual in the model with individual-specific random error ($\sigma ^{2}_{l}=\sigma ^{2}_{i}$):

4 $$ {}\begin{aligned} LL_{i}&=\sum_{j=1}^{n_{i}}\left\{-\frac{1}{2}ln(2\pi\sigma_{i}^{2})-\frac{1}{2\sigma_{i}^{2}}\left(y_{ij}-f(t_{ij})\right.\right.\\ &\quad\qquad\left.-\beta_{1}'x_{1i}-a_{0i}-a_{1i}t_{ij})^{2}{\vphantom{\frac{1}{2}}}\right\} \\ &\quad-I_{Ri}log\left\{1\,-\,F_{i}(t_{Ri})\right\}+(1\,-\,I_{Ri})log\left\{F_{i}(t_{Ri})\,-\,F_{i}(t_{Li})\right\},  \end{aligned}  $$

where *I*_*Ri*_ is an indicator that the event for individual *i* is right censored (1=right censored, 0=interval censored), and *F*_*i*_(*t*) is the cumulative density function at time t.

### Model specifications

Four models were fit to test the clinical hypothesis that the variability of the biomarker over time would be associated with the time to event outcome. These are all special cases of the joint model outlined in “General model formulation” section.

- Model 1 (M1): Base model. *l*=1, so all patients have a common residual error, and *g*_*i*_=0, so there are only two shared parameters: the random intercept and slope.
- Model 2 (M2): Individual-specific variance model. *l*=*i*, so each patient is allowed a different residual error term, and *g*_*i*_=0, so again, there are only two shared parameters: the random intercept and slope.
- Model 3 (M3): Individual-specific variance shared model. *l*=*i*, so each patient is allowed a different residual error, and *g*_*i*_=*σ*_*i*_, so *λ*_2_ represents the hazard of event associated with the residual standard error.
- Model 4 (M4): Individual-specific coefficient of variation (CV) shared model. *l*=*i*, so each patient is allowed a different residual error, and $g_{i}=\frac {\sigma _{i} }{\frac {1}{n_{i}}\sum _{j=1}^{n_{i}}{y}_{ij}}$, so *λ*_2_ represents the hazard of event associated with the coefficient of variation. Of note, the CV is calculated here as the estimated standard deviation over the sample mean.

### Model selection and predictive ability

The Deviance Information Criterion (DIC) [13] and the Watanabe-Akaike information criterion (WAIC) [14] were used for model selection. The DIC is calculated based on the deviance, *D*(***θ***)=−2 log*L*(***y***|***θ***) and penalizes for the number of effective degrees of freedom in the model. WAIC approximates leave-one-out cross-validation [14] and works well for hierarchical models where the number of degrees of freedom is not obvious [15]. The best model should have the lowest values of DIC and WAIC; differences of at least 5 points were considered important.

The predictive ability of the four models was assessed using Area Under the Curve (AUC) and Brier Score (BS). The interval censored nature of dnDSA complicates the calculation of AUC and BS. In right-censored survival settings, the AUC is the proportion of concordant pairs among all subject pairs, where the pair achieves concordance if the risk of event is higher for the subject with the earlier event time [16]. Under interval censoring, it may not be clear which subject in the pair has the earlier event time. When a parametric baseline hazard is employed, such as the Weibull, various methods for calculating the probability that the event time of the first subject is less than the event time of the second have been proposed [17, 18]. We used both the nonparametric estimator approach proposed by Wu and Cook and the probability based methods outlined by Tsouprou to calculate AUC, and only reported the Tsouprou results as the two both showed the same conclusions. BS is defined as the mean squared difference between the predicted probability and the observed outcome for each subject at a pre-specified follow-up time. Due to the interval censored nature of the data, is not always known if the outcome occurred before a certain time, so again we calculate the probability that the patient had the event prior to a pre-specified time [17].

Due to the time-varying nature of the models, the AUC and BS can change over time. To assess the predictive ability of the models at differing time points for differing prediction windows, we chose two clinically relevant scenarios: (1) being dnDSA-free at 12 months, given the patient was dnDSA-free at 6 months, and (2) being dnDSA-free at 24 months, given the patient was dnDSA-free at 12 months.

### Dynamic prediction of survival probabilities

In order to compare predictive abilities of the proposed models (M1-M4), we calculate the dynamic predictions of surviving dnDSA-free at a pre-specified time point. Several works show the derivation of the conditional predictive probability of survival for a new individual at time *t*^′^, given that the individual has survived up to time *t*, where *t*^′^>*t* [19, 20]. Let $y^{t}_{j} $ denote the longitudinal trajectory up to time *t*, and *T*^∗^>*t* denote that the new individual has survived up to time *t*. An important step of this calculation is obtaining samples of the conditional posterior distribution of the random effects for this new individual $a_{N} \left (a_{N}=(a_{N0}, a_{N1})'\right), p\left (a_{N} \mid T^{*} >t, y^{t}_{j}, \theta \right)$, where *θ* denotes the set of all parameter values of the model. Conditional on the *m*-th MCMC sample *θ*^*m*^, we draw the *m*-th sample of the random effects vector from its posterior distribution

5 $$ {}\begin{aligned} p\!\left(a_{N} \!\mid T^{*} \!>\!t, y^{t}_{j}, \theta^{m}\!\right)&\,=\, p\left(y^{t}_{j} \mid \theta^{m}, a_{N}\!\right) p\left(T^{*} >t \mid \theta^{m}, a_{N}\right)\\ &\quad\ p\left(a_{N} \mid \theta^{m}\right), \end{aligned}  $$

where $p\left (y^{t}_{j} \mid \theta ^{m}, a_{N}\right)$ and *p*(*T*^∗^>*t*∣*θ*^*m*^,*a*_*N*_) are the conditional probabilities of the longitudinal and survival outcomes, and *p*(*a*_*N*_∣*θ*^*m*^) is the probability of the random effects vector. We follow ideas in [20] and employ adaptive rejection Metropolis Hastings sampling to draw samples of the random effect (5) and calculate the predicted survival probabilities from the MCMC samples.

Survival probabilities may be obtained using any time points (*t*^′^ and *t*, *t*^′^>*t*), but for simplicity and for clinical applicability, we chose to assess two scenarios: (1) being dnDSA-free at 12 months, given the patient was dnDSA-free at 6 months (*t*=6,*t*^′^=12), and (2) being dnDSA-free at 24 months, given the patient was dnDSA-free at 12 months (*t*=12,*t*^′^=24). Oftentimes, the patient comes to clinic for a milestone appointment (say 1 year-post transplant) appointment, and the clinician wants to assess the probability of the patient continuing to remain dnDSA-free until the next appointment (at 2 years post-transplant, for example). We used this framework to assess and compare the Area Under the Curve (AUC) and Brier Score (BS) for each model under each of the time period scenarios. For particular patients, we also calculated the dynamic predictions at more time points, specifically where *t* was 12 months and *t*^′^ ranged from 18 months to 60 months, to demonstrate how the survival probabilities can be calculated for longer follow-up periods.

### Application

Five hundred and thirty eight patients met the final inclusion criteria for this analysis. The dataset was randomly split into a training (2/3, N=358) and a testing (1/3, N=180) cohort. The four models outlined in the methods were fit to the training cohort (N=358). Each model had a longitudinal sub-model (Eq. 1) with TAC as the outcome, a fixed intercept and slope for time (*f*(*t*_*ij*_)=*b*_0_+*b*_1_*t*_*ij*_)) and a random intercept and slope for time for variations across individuals. Spline terms for time were tested, but DIC and WAIC indicated that a linear trend was sufficient. For the survival sub-models (Eq. 3), various baseline hazard functions were tested, including exponential, Weibull, gamma, and piecewise constant, and the Weibull yielded the best model fit (*h*_0_(*t*)=*αt*^*α*−1^, where *α* is the Weibull shape parameter). All survival sub-models had time to dnDSA as the interval censored outcome, random effects associated with the longitudinal sub-model and a vector of baseline covariates for age (younger age: <30, middle age: 30 −49, older age: 50 +), race/ethnicity (Caucasian, African American, Hispanic, and Other), and number of HLA mismatches. More detail on each of these characteristics and why they are hypothesized to be associated with dnDSA can be found elsewhere [6]. All models built upon the base model, M1, which had a shared random intercept and slope. As described in “Model specifications” section, M2 allowed each subject to have an individual-specific residual error term, M3 shared this individual-specific error term with the hazard sub-model, and M4 shared the individual-specific coefficient of variation with the hazard sub-model. Thus, the final implemented models followed this general structure:

6 $$ TAC_{ij}\sim N\left(\mu_{ij}, \sigma^{2}_{l}\right), \text{where}\ \mu_{ij}=b_{0}+b_{1}t_{ij}+a_{0i}+a_{1i}t_{ij},   $$

7 $$ \begin{aligned} h_{i}(t)\!&=\!\alpha t^{\alpha-1}\!\exp(\beta_{0}\,+\,\beta_{1}\textrm{HLA}\,+\,\beta_{2}\textrm{African American}\\&\quad+\beta_{3}\text{Hispanic}+ \beta_{4}\textrm{Other Race}\\&\quad+\beta_{5}\textrm{Middle Age}+\beta_{6}\textrm{Older Age}\\&\quad+ \lambda_{0} a_{0i}+\lambda_{1} a_{1i}+\lambda_{2}g_{i}).  \end{aligned}  $$

Model estimation and inference was based on the Bayesian framework. Hyper-parameters are assigned weakly informative priors:

$$\begin{array}{*{20}l} b_{0},b_{1},\beta_{0},\beta_{1}',\beta_{2}',\lambda_{0},\lambda_{1},\lambda_{2}, & \stackrel{iid}{\sim} N(0,10000) \\ log(\sigma_{l}) &\sim \text{Uniform}(-100,100) \\ \alpha&\sim \text{Gamma}(100,100) \\ \left[\begin{array}{cc} \sigma_{0}^{2} & \rho\sigma_{0}\sigma_{1} \\ \rho\sigma_{0}\sigma_{1} & \sigma_{1}^{2} \\ \end{array}\right]^{-1} & \sim \text{Wishart}\left(\begin{array}{ll} \left[\begin{array}{cc} 0.00001 & 0 \\ 0 & 0.000001 \\ \end{array}\right] \end{array},2\right). \end{array} $$

Denoting *u* as a generic random variable, we define the following density functions: *u*∼Normal(*μ*,*σ*^2^) with probability density $1/(\sigma \sqrt {2\pi })\exp {\{-(u-\mu)^{2}/2\sigma ^{2}\}}$; *u*∼Uniform(*a*,*b*) with probability density 1/(*b*−*a*) for *a*<*u*<*b*; *u*∼Gamma(*r*,*λ*) with probability density *λ*^*r*^*u*^*r*−1^exp(−*λu*)/ *Γ*(*r*); and *Ω*∼Wishart(*R*,*k*) with probability density (∣*Ω*∣^(*k*−*p*−1)/2^∣*R*∣^*k*/2^ exp{−*Tr*(*RΩ*/2})/(2^*pk*/2^*Γ*_*p*_(*k*/2)) for *k*≥*p*, where *p*=2, *Tr* is the trace function and *Γ*_*p*_ is the multivariate gamma function. Notably, we defined the prior for log(*σ*_*l*_) as uniform(-A, A), where A is sufficiently large (we used A=100).

The posterior distribution of each variable was estimated using Markov Chain Monte Carlo (MCMC) simulations. A Gibbs sampler was used to form two Markov chains using JAGS software [21] and the runjags package in R [22]. Convergence of the samples was assessed by trace plot inspection, and Gelman and Geweke tests which test for equality of the means of the first and last part of a Markov chain [23]. After a burn-in period of 20,000 and thinning of 50 of 100,000 sampling iterations, 2,000 samples per chain were used for inference. On average, each model took approximately 3.5 hours to run on a PC with two Intel Xeon X5650 processors and 128 GB RAM. For dynamic prediction, 500 MCMC samples were used, and within each sample, 500 iterations of the Metropolis Hastings algorithm were employed to estimate the random effects. Calculating a prediction for one subject took approximately 40 minutes on the same PC, using parallel processing on 12 cores. A reproducible example of M4, including code to fit the model and calculate dynamic predictions, can be found in the Supplemental Materials.

## Results

**Fig. 1:**
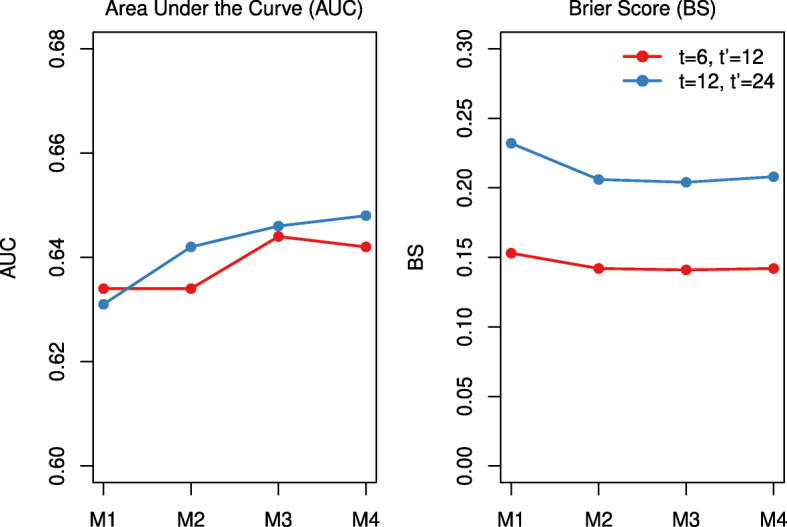
Comparison of Area under the Curve (AUC) and Brier Score (BS) for each of the four models, under two scnearios: (1) being dnDSA-free at 12 months, given the patient was dnDSA-free at 6 months, and (2) being dnDSA-free at 24 months, given the patient was dnDSA-free at 12 months. M1=base model, shared random intercept and slope only, M2=allow for individual specific random error term, M3=share the individual specific standard deviation with hazard, M4=share the individual specific CV with hazard

A comparison of WAIC and DIC for each model, M1-M4, is reported in Table 1. As shown by lower DIC and WAIC, all three models that allowed for individual-specific variation (M2, M3, M4), performed better than the base model that forced a common residual error term. The model that fit the data best according to DIC allowed for individual-specific residual error and shared the CV term with the hazard of event. M2 had the lowest WAIC. M2-M4 also had superior predictive ability of dnDSA-free survival compared to M1, demonstrated by higher AUC and lower BS (Fig. 1 and Table S1).

**Table 1:** Model selection criteria: DIC, and WAIC as defined in the Model selection and predictive ability section

Model	DIC	WAIC
M1: Shared random intercept and slope only	46022	46137
M2: Individual variance term (not shared)	44580	44765
M3: Shared individual variance term	44587	44791
M4: Shared individual CV term	44571	44776

Training cohort, N=358

**Fig. 2:**
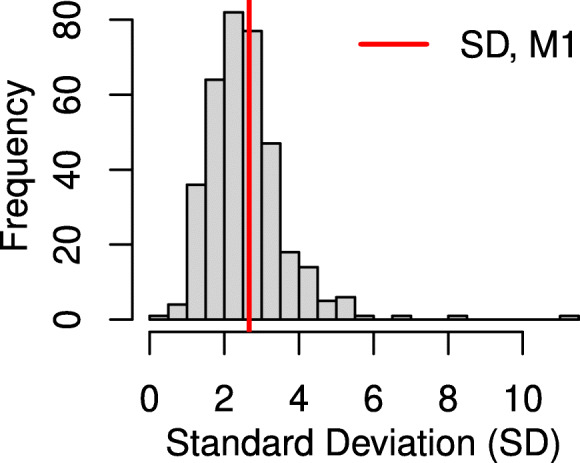
Model 4 (M4) allowed each individual to have it’s own random error term, to account for varying amounts of variability in TAC between patients. The distribution of the individual’s standard deviations (SD) is shown here as a histogram. The red vertical line represents the common standard deviation taken from M1, which forced each individual to have a common residual error

The model estimates are found in Table 2. The resulting estimates for the TAC sub-model (fixed intercept, slope, covariance matrix of random intercept and slope) are similar across the M1-M4. M1 has a common random error term for all patients, which was estimated as 7.15 (95% Credible Interval (CrI): 6.93, 7.36). All other models allowed for residual error to vary by patient, and the estimated standard deviations from M4 ranged from 0.46 to 11.16 (Fig. 2). The hazard ratios for all baseline covariates (*β*_0_−*β*_6_) are similar in magnitude across the models. The loading parameters for the shared random effects (*λ*_0_,*λ*_1_) are similar across the models, although the *λ*_1_ is slightly lower for M1 compared to the other three. *λ*_2_ represents the effect the individual-specific residual standard deviation (M3) and coefficient of variation (M4) on the hazard of dnDSA. For every one unit increase in an individual’s estimated residual standard deviation, the risk of dnDSA increases 1.25-fold (95% CrI: 1.00, 1.54), and for every one unit increase in an individual’s estimated CV, risk of dnDSA increases 1.40-fold (95% CrI: 0.12, 5.03).

**Table 2:** Results from all models

Parameter	Model 1	Model 2	Model 3	Model 4
Linear Sub-Model	Estimate (95% CrI)	Estimate (95% CrI)	Estimate (95% CrI)	Estimate (95% CrI)
*b*_1_	-0.059 (-0.071, -0.047)	-0.056 (-0.068, -0.044)	-0.056 (-0.068, -0.044)	-0.056 (-0.069, -0.045)
*b*_0_	7.254 (7.094, 7.411)	7.177 (7.007, 7.324)	7.182 (7.014, 7.338)	7.178 (7.027, 7.32)
*ρ*	-0.419 (-0.562, -0.264)	-0.427 (-0.564, -0.275)	-0.422 (-0.555, -0.268)	-0.424 (-0.563, -0.268)
$\sigma ^{2}_{0}$	1.769 (1.455, 2.124)	1.716 (1.42, 2.074)	1.711 (1.394, 2.069)	1.713 (1.397, 2.072)
$\sigma ^{2}_{1}$	0.005 (0.003, 0.007)	0.005 (0.003, 0.006)	0.005 (0.003, 0.007)	0.005 (0.003, 0.007)
$\sigma ^{2}_{e}$	7.146 (6.931, 7.359)	NA (NA, NA)	NA (NA, NA)	NA (NA, NA)
Survival Sub-Model	Hazard Ratio (95% CrI)	Hazard Ratio (95% CrI)	Hazard Ratio (95% CrI)	Hazard Ratio (95% CrI)
*α*	0.555 (0.439, 0.698)	0.514 (0.413, 0.623)	0.535 (0.426, 0.666)	0.524 (0.421, 0.647)
*β*_0_	0.035 (0.012, 0.075)	0.043 (0.018, 0.085)	0.024 (0.006, 0.057)	0.045 (0.014, 0.107)
*β*_1_ (HLA Mismatch Number)	1.262 (1.115, 1.437)	1.239 (1.098, 1.393)	1.236 (1.086, 1.394)	1.238 (1.100, 1.403)
*β*_2_ (African American)	2.015 (1.003, 3.644)	1.612 (0.857, 2.717)	1.673 (0.872, 2.805)	1.634 (0.857, 2.794)
*β*_3_ (Hispanic)	1.466 (0.842, 2.324)	1.54 (0.919, 2.365)	1.398 (0.836, 2.232)	1.540 (0.899, 2.420)
*β*_4_ (Other Race)	1.092 (0.21, 2.909)	0.949 (0.192, 2.465)	0.944 (0.168, 2.459)	0.951 (0.189, 2.484)
*β*_5_ (Middle Age)	0.721 (0.35, 1.37)	0.803 (0.41, 1.482)	0.784 (0.398, 1.463)	0.802 (0.405, 1.472)
*β*_6_ (Older Age)	0.307 (0.137, 0.616)	0.34 (0.168, 0.627)	0.338 (0.163, 0.655)	0.335 (0.165, 0.623)
*λ*_0_	0.659 (0.515, 0.813)	0.66 (0.539, 0.802)	0.644 (0.517, 0.78)	0.661 (0.491, 0.845)
*λ*_1_ (per 0.05 change)	0.478 (0.315, 0.646)	0.578 (0.441, 0.731)	0.559 (0.41, 0.714)	0.569 (0.410, 0.749)
*λ*_2_	NA (NA, NA)	NA (NA, NA)	1.254 (1.004, 1.543)	1.400 (0.119, 5.026)

The posterior mean and 95% credible intervals are presented for the linear portion of the model, the survival portion of the model, and the association parameters. The longitudinal portion is comprised of a fixed intercept (*b*_0_), a fixed slope (*b*_1_), and a random error term ($\sim N(0,\sigma ^{2}_{e})$). In Models 2-4, the random error term is individual specific ($\sim N(0,\sigma ^{2}_{ie})$) There is also a random intercept ($a_{0i}\sim N(0,\sigma ^{2}_{0})$) and a random slope for each individual ($a_{1i}\sim N(0,\sigma ^{2}_{1})$), which have a correlation parameter *ρ*. The survival model is comprised of fixed covariates (*β*), the Weibull association parameter *α*, and a random intercept for each individual that is related to the random intercept, slope, and some function of the estimated longitudinal trajecotry, through association parameters. The first association parameter (*λ*_0_) links the two sub-models through their shared random intercepts. The second association parameter (*λ*_1_) links the two sub-models through the longitudinal random slope and the survival random intercept. In Models 2-4, *λ*_2_ links the two sub-models through the longitudinal individual-specific SD (Model 3) or CV (Model 4) term. Middle age=30-49 years, Older age=50+ years. Model 1 (M1)=base model, shared random intercept and slope only, Model 2 (M2)=allow for individual specific random error term, Model 3 (M3)=share the individual specific standard deviation with hazard, Model 4 (M4)=share the individual specific CV with hazard. Training cohort, N=358

**Fig. 3:**
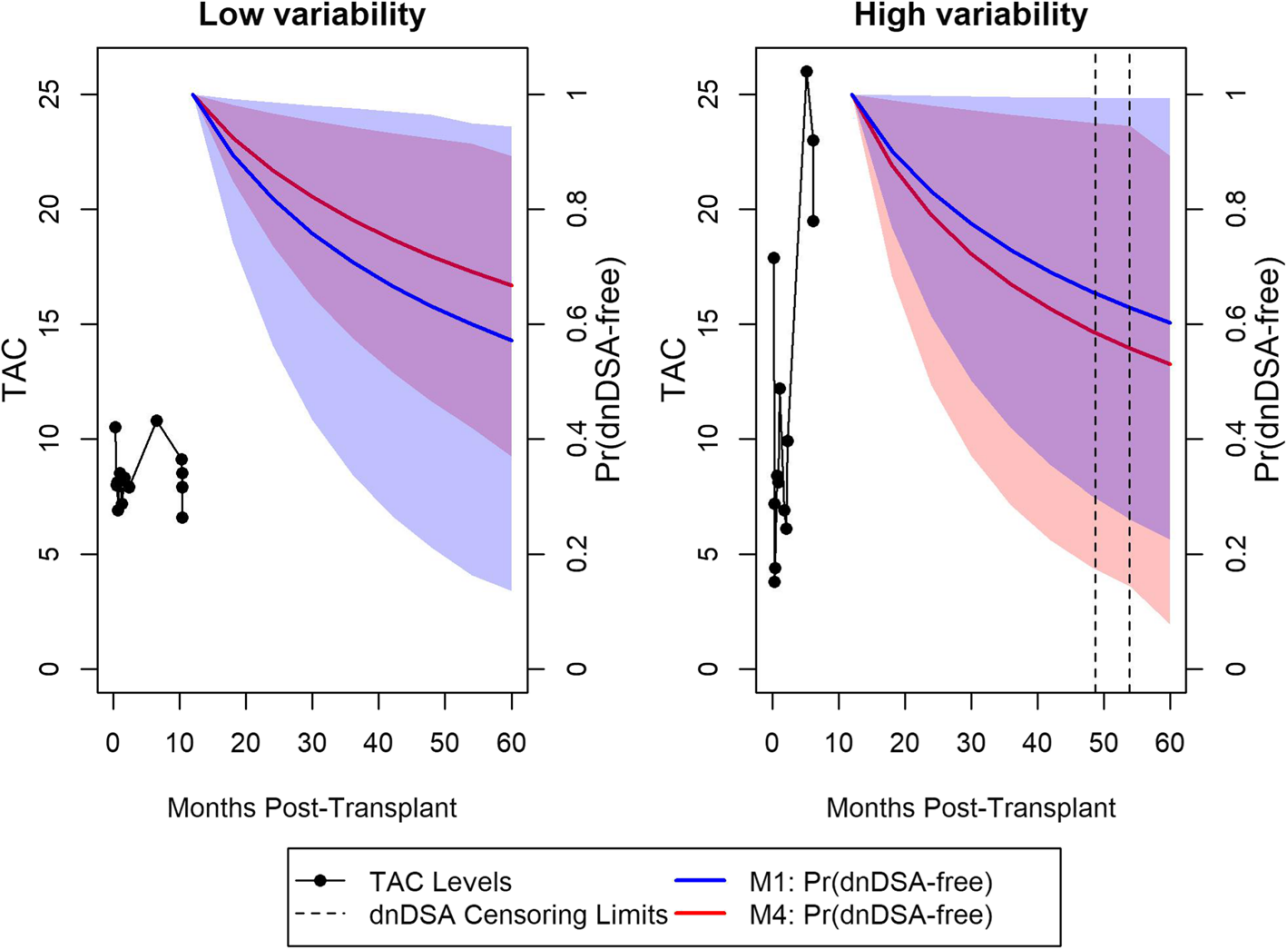
Predicted probability of dnDSA-free survival, conditional on survival to 12 months. Left-hand panel: black lines represent observed TAC values for a given individual with good adherence (low coefficient of variation) of TAC during the first year post-transplant. Red curve represents predicted probability from M4 (model with coefficient of variation shared parameter) of remaining dnDSA-free up to 5 years post-transplant. Blue curve represents predicted probability from M1 (base model, does not account for variability) of remaining dnDSA-free up to 5 years post-transplant. The 95% credible intervals for the predictions are indicated by the shaded regions. The right-hand figure is the same framework, but for a patient with bad TAC adherence, and who developed dnDSA sometime between 48-55 months post-transplant

Dynamic predictions for experiencing dnDSA-free survival were calculated for two individuals in Fig. 3. Both individuals were dnDSA-free at 12 months, and one went on to experience a dnDSA event, while the other did not. The probability of surviving dnDSA-free from 12 months to 60 months, by an increment of 6 months, was calculated for each individual, separately using the output from M1 and M4. In the left-hand panel is an individual who had a low CV of TAC during the first year post-transplant. This patient did not go on to experience a dnDSA event within 5 years post-transplant, and the estimated probability of dnDSA-free survival is higher using M4 compared to M1. In the right-hand panel, an individual who had a high CV of TAC during the first year post-transplant is presented. This patient went on to develop TAC sometime between 48 and 54 months post-transplant, and the patient’s probability of dnDSA-free survival is lower using M4, compared to M1. For both of these individuals, M4 yielded a better prediction of dnDSA compared to M1.

### Simulation study

We conducted a simulation study to evaluate the performance of a model that allowed for individual-specific variances on a finite sample. We used M4 to simulate data, as this was the model with the most complexity. The true values of each parameter, listed in Table 3, were set to be close to the resulting estimates from the training dataset. Simulation results were obtained from 200 datasets, each with 200 individuals. Models were run for 1,000 iterations, after a 1,000 iteration burn-in time. As shown in Table 3, all empirical means (means of the 200 estimates) are close to the true values, except there was a small bias in *α*, the shape of the Weibull baseline hazard function, and in *λ*_2_, the association parameter for *g*_*i*_, the individual-specific CV.

**Table 3:** Simulation study results of M4, model with shared individual-specific CV parameter (data simulated using M4, 200 datasets of N=200)

Parameter	True Value	Mean	SD
*b*_1_	-0.03	-0.029	0.008
*b*_0_	7	7.006	0.102
*ρ*	-0.03	-0.001	0.088
$\sigma ^{2}_{0}$	1.75	1.769	0.204
$\sigma ^{2}_{1}$	0.004	0.004	0.001
*β*_0_	-2	-2.006	0.344
*β*_1_ (HLA Mismatch Number)	0.25	0.265	0.085
*α*	0.5	0.516	0.04
*λ*_0_	-0.5	-0.551	0.289
*λ*_2_ (loading parameter for individual-specific CV)	0.2	-0.179	0.480

## Discussion

Tacrolimus is the most important immunosuppressant drug in solid organ transplantation despite having a very narrow therapeutic window. Ensuring appropriate drug exposure within this window is critical in order to maintain health of the graft while avoiding drug toxicities. However, understanding intrapatient drug exposure has been challenging, as only using average drug levels, or one-dimensional summary statistics of drug levels, has several disadvantages that don’t represent a complete picture of TAC exposure over time. The variability of TAC, using all longitudinal measures, may be a useful way to help characterize TAC drug exposure, as variability often leads to more time out of therapeutic range and places patient at risk for adverse outcomes [6]. In this paper, we demonstrated that incorporating an individual-specific variance term into the modeling of TAC and dnDSA indeed improves model fit and prediction of dnDSA.

The random effects in the joint model induce the dependence between the two sub-models, which allows us to test whether the variance and CV of longitudinal TAC is associated with time-to-dnDSA, while accounting for the unobserved heterogeneity in these functions. Unlike the random effects, the biomarker’s variability (embodied in the CV) has an intuitive clinical interpretation. In our joint model context, its contribution is readily quantified as a hazard of experiencing a dnDSA event. If we observe two patients that only differ in the CV of TAC by one unit, everything else being equal for a specific time, the hazard of dnDSA is 1.4-times higher for the individual with the larger CV. Similarly, if two patients differ only in the estimated random error of TAC by 1 unit, the hazard of dnDSA is 1.3-fold higher for the patient with the higher variability.

The number of variance parameters estimated by M2-M4 increases as the sample size increases. These exchangeable random-effects arise naturally in Bayesian hierarchical modeling, enabling a flexible individual-level structure of dependence, and computationally they are very convenient. The estimates at the individual-level shrink towards a common population-level estimate, which intuitively allows for better modeling of any individual’s longitudinal trajectory by borrowing information from similar subjects. In other words, exchangeability allows the use of information from the entire cohort to strengthen the inference for any individual subject. In Bayesian jargon, this is called “borrowing strength” [24]. The DIC and WAIC metrics both account for the effective degrees of freedom, particularly important in models with complex hierarchies of random effects; and despite additional parameters, the models with individual-specific variances proved to be superior to the model with homogeneous variance (Table 1). Additionally, the individual-level parameters allow individual-specific estimation, improving the dynamic predictions (as described in “Dynamic prediction of survival probabilities” section).

The models with heterogeneous variance (M2-M4) proved to have superior predictive performance than M1, as shown by AUC and BS. The AUC was generally higher for predicting at *t*=12 months, compared to *t*=6 months, perhaps because we have more available longitudinal measurements to use in the prediction at the later time point. The BS was lower for the scenario of *t*=6 and *t*^′^=12, compared to the scenario of *t*=12 and *t*^′^=24, perhaps because the window of prediction was shorter (6 versus 12 months). Dynamic predictions using this model may be employed as a tool of personalized medicine, providing a guide for the clinical decisions, e.g. by modifying interventions or acting as alerts in responding to the longitudinal profile of the patient. As seen in Fig. 3, incorporating the variability in to the model improved the predictions for these two individuals, which highlights the need to account for the overall trajectory and variability of TAC when assessing risk of dnDSA. Models such as these could be applied to any disease that is treated with medications that have a narrow therapeutic window that require drug monitoring, such as glomerularnephritides, autoimmune conditions, and some cancers.

We have employed some of the most used parameterizations of joint models (e.g. sharing the random effects) as well as one that is less common, which considers the sharing of other characteristics of the longitudinal trajectory, namely the standard deviation of the residual error. In future work, we plan to explore alternatives to JAGS, such as Nimble [25] or Stan [26], as these approaches may speed up the computational time needed to perform the dynamic predictions. Another natural extension of this work would be to adopt the idea of the latent class joint models, which could be used to identify the patients whose prognostic can be improved by adjusting the dose of TAC and patients for whom the dose adjustment will fail. After model adjustment, subjects of different latent classes can be easily interpreted with survival probabilities plots, thus providing a good prognostic tool.

## Conclusion

Using a joint model with a flexible linkage function, we demonstrated that an individual’s variability of TAC over time is important in dynamically predicting dnDSA post kidney transplant. The proposed model improved prediction of dnDSA over a method that did not model the variability in TAC, and has potential of improving clinical outcomes in this area of personalized medicine.

## Supplementary Information

**Additional file 1** Dynamic prediction based on variability of a longitudinal biomarker Supplemental Materials

## Data Availability

The datasets generated and/or analysed during the current study are not publicly available due to being owned by the University of Colorado Transplant Center and containing protected health information, but are available from the corresponding author on reasonable request. Declarations

## Abbreviations

TAC: Tacrolimus
dnDSA: De novo donor specific antibody
JM: Joint model
MCMC: Markov Chain Monte Carlo
CrI: Credible interval
HLA: Human leucocyte antigen
JAGS: Just another Gibbs sampler
DIC: Deviance information criterion
WAIC: Watanabe-Akaike information criterion
IC: Interval censoring
