# Supplementary material for: Dynamic prediction based on variability of a longitudinal biomarker
Source: BMC Med Res Methodol. 2021 May 15;21:104. doi: 10.1186/s12874-021-01294-x (PMC8122571; doi:10.1186/s12874-021-01294-x)
Supplement: Supplementary file 1 — Additional file 1 Dynamic prediction based on variability of a longitudinal biomarker Supplemental Materials [file 12874_2021_1294_MOESM1_ESM.pdf]

Dynamic prediction based on variability of a longitudinal biomarker Supplemental Materials

March 19, 2021

Table S1: Comparison of Area under the Curve (AUC) and Brier Score (BS) for each of the four models, under two scenarios: (1) being dnDSA-free at 12 months, given the patient was dnDSA-free at 6 months, and (2) being dnDSA-free at 24 months, given the patient was dnDSA-free at 12 months.

	t: 12, t': 24	t: 12, t': 24	t: 6, t': 12	t: 6, t': 12
Model	AUC	BS	AUC	BS
M1: Shared random intercept and slope only	0.631	0.232	0.634	0.153
M2: Individual variance term (not shared)	0.642	0.206	0.634	0.142
M3: Shared individual variance term	0.646	0.204	0.644	0.141
M4: Shared individual CV term	0.648	0.208	0.642	0.142

M4 JAGS Model

```
##############################################################
#  M4 joint model: survival and Linear mixed model:
#  Shared random intercept, slope, and CV
##############################################################

##### Joint Model : shared random slope with random int
model {
mu.re[1] <- 0
mu.re[2] <- 0
### linear model
for(i in 1:N){
for(j in 1:k[i]){
Y[i,j] ~ dnorm(true.y[i,j], tauy[i])
true.y[i,j] <- m1 * (X[i,j]) + c + a1[i]+a2[i]*X[i,j]
log_lik01[i,j] <- (- log(tauy[i]) + log(2*3.14159)  +
pow(Y[i,j]-true.y[i,j],2) * tauy[i])/(-2)
}
## random effects
re[i,1:2] ~ dmnorm(mu.re[1:2],omega.bc[,]);  # bivariate Normal
a1[i] <- re[i,1]
a2[i] <- re[i,2]
#log likelihood for linear model:
ll_l[i] <- sum(log_lik01[i,1:k[i]])
### survival model
true.mean[i]<-mean(Y[i,1:k[i]])
cv[i]<-sigmay[i]/true.mean[i]

log(mu[i]) <- b2+b_hla_s*hla[i]+b_aa_s*aa[i]+b_hisp_s*hisp[i]+b_other_s*other[i]+
b_mid_s*mid[i]+b_old_s*old[i]
+ga1*a1[i[i]]+ga2*a2[i[i]]+ga3*cv[i]

ind.i[i] ~ dinterval(t[i], lim[i,])
t[i] ~ dweib(alpha, mu[i])
##log likelihood: survival model
ll_s[i] <- (ind.i[i]-1)*log(1-pweib(lim[i,2],alpha, mu[i])) +
(2-ind.i[i])*log(pweib(lim[i,2],alpha, mu[i])- pweib(lim[i,1],alpha, mu[i]))
}

## priors survival model
b2~dnorm(0,0.0001);
b_hla_s~dnorm(0,0.0001);
b_aa_s~dnorm(0,0.0001);
b_hisp_s~dnorm(0,0.0001);
```

```
b_other_s~dnorm(0,0.0001);
b_mid_s~dnorm(0,0.0001);
b_old_s~dnorm(0,0.0001);
ga1~dnorm(0,0.0001);
ga2~dnorm(0,0.0001);
ga3~dnorm(0,0.0001);
alpha~dgamma(0.01,0.01)

## priors linear model
m1 ~ dnorm(0,0.0001);
c ~ dnorm(0,0.0001);
sige~dunif(0,100);

#parameters considered MVN
omega.bc[1:2,1:2]  ~  dwish(Rom,2);    # Wishart prior on precision matrix
sig2.bc[1:2,1:2] <-inverse(omega.bc[1:2,1:2]);
sig.bc[1] <- sig2.bc[1,1];
sig.bc[2] <- sig2.bc[2,2];
rho.0r <- sig2.bc[1,2] / (sqrt(sig2.bc[1,1])*
sqrt(sig2.bc[2,2]));  # correlation intercept and slope

##new variance:
for(i in 1:N){
log.sigmay[i] ~ dunif(-100,100)
sigmay2[i] <- pow(exp(log.sigmay[i]), 2)
tauy[i] <- 1/sigmay2[i]
sigmay[i] <- sqrt(sigmay2[i]) # std. deviation of the longitudinal measures
}
}
```
